# A Novel Perturbed Spiral Sheathless Chip for Particle Separation Based on Traveling Surface Acoustic Waves (TSAW)

**DOI:** 10.3390/bios12050325

**Published:** 2022-05-11

**Authors:** Miaomiao Ji, Yukai Liu, Junping Duan, Wenxuan Zang, Yongsheng Wang, Zeng Qu, Binzhen Zhang

**Affiliations:** 1 Key Laboratory of Instrumentation Science & Dynamic Measurement, Ministry of Education, North University of China, Taiyuan 030051, China; s2006240@st.nuc.edu.cn (M.J.); duanjunping@nuc.edu.cn (J.D.); s1906173@st.nuc.edu.cn (W.Z.); s202106004@st.nuc.edu.cn (Y.W.); 20210057@nuc.edu.cn (Z.Q.); 2 Science and Technology on Electronic Test and Measurement Laboratory, North University of China, Taiyuan 030051, China; s2006073@st.nuc.edu.cn

**Keywords:** spiral channel, micropillar array, slanted gold IDT, particle separation, microfluidics

## Abstract

The combination of the new perturbed spiral channel and a slanted gold interfingered transducer (IDT) is designed to achieve precise dynamic separation of target particles (20 μm). The offset micropillar array solves the defect that the high-width flow (avoiding the occurrence of channel blockage) channel cannot realize the focusing of small particles (5 μm, 10 μm). The relationship between the maximum design gap of the micropillar (Smax) and the particle radius (*a*) is given: Smax = 4*a*, which not only ensures that small particles will not pass through the micropillar gap, but also is compatible with the appropriate flow rates. A non-offset micropillar array was used to remove 20 μm particles in the corner area. The innovation of a spiral channel structure greatly improves the separation efficiency and purity of the separation chip. The separation chip designed by us achieves deflection separation of 20 μm particles at 24.95–41.58 MHz (*κ* = 1.09–1.81), at a flow rate of 1.2 mL per hour. When *f* = 33.7 MHz (*κ* = 1.47), the transverse migration distance of 20 μm particles is the smallest, and the separation purity and efficiency are as high as 92% and 100%, respectively.

## 1. Introduction

Fluorescence-activated cell sorting machines (FACS) are traditional cell sorting tools which have been widely used in biomedical and clinical protein experiments [1,2,3]. However, there are still some disadvantages, such as high cost, large volume, and difficulty handling small samples. Compared to FACS, microfluidic devices require only a small sample volume to achieve the yield achieved by FACS with a sample volume several orders of magnitude larger. Microfluidic fluorescence-activated cell sorters (μFACS) overcome the limitation that FACS cannot handle small samples.

Over the years, microfluidic devices have made great progress in the application of cell sorting. Microfluidic cell sorting equipment has developed from passive sorting [4,5,6] to active sorting [7,8,9]. The former relies on the geometrical shape of the channel and the intrinsic hydrodynamic force, which develops the particle focusing element based on hydrodynamics and inertia; after the focusing unit, the sorting is realized according to the different focusing positions of different particles [10]. The latter usually allows for more precise control of target particles. Cell sorting units driven by dielectric electrophoresis [11] (DEP), surface acoustic wave [12] (SAW), and optical tweezers [13] were developed. Acoustic control particles have been widely studied and concerned due to their advantages of non-contact, good biological compatibility, no need for chemical biomarkers on the particles, and easy integration of devices.

Particles are arranged before acoustic manipulation. One method is to bind particles in the middle of the flow channel by adding sheath flows on both sides [14]. The other uses inertial focusing to focus particles into a line or form a particle beam [15]. The former requires multiple pumps and a large amount of sheath fluid, which may cause contamination due to its flow. The latter overcomes the limitations described in the former, so the sheathless chip design is favored by researchers.

In the acoustic control area, due to the different electrode design and arrangements, there are two main control modes: standing surface acoustic waves (SSAW) control [16,17,18] and TSAW control [19,20,21]. Although the former can accurately control the horizontal displacement, at least two IDTs need to be placed in pairs, so the system is complex and the operation is somewhat challenging. Compared with the former, the latter system and operation is relatively simple. Guojun Liu et al. designed a focused sound separation chip based on TSAW separation mechanism [21]. Finally, the chip can achieve the separation of 1 μm and 10 μm polystyrene particles, and the separation efficiency is more than 99%. The separation of various particle sizes is not reflected in their work, which leads to some limitations in their application. K. Mutafopulos et al. sorted cells by TSAW after combining inertial flow focusing with sheath flow [15], which can be used to classify three different cell lines with a purity of about 90%. However, both of these designs require the introduction of sheath flow, which increases the cost of testing equipment and the possibility of sample contamination. Therefore, the design and preparation of a sheathless TSAW chip that can separate various particle sizes is expected by researchers.

In this paper, we report a microfluidic device based on TSAW. The device combines a spiral channel for focusing large particles and a slanted gold IDT. The spiral channel can focus the large particles in the center of the flow channel, while the small particles cannot form the focusing streamline. In order to improve this phenomenon, we have made two improvements to the spiral chip. Firstly, particles of all sizes were collected in the area 150 μm away from the outer wall of the spiral channel by setting an offset micropillar array in the flow channel before the outlets. Secondly, a non-offset micropillar array is designed on the outer wall before the outlets to avoid the target particles entering the corner effect region. The above two improvements significantly improve the separation efficiency and purity of the chip. By exciting TSAW pulses at different frequencies, large particles can achieve different degrees of deflection into the target export, while other small particles keep their original trajectory and exit through the non-target export. Compared with the sheath TSAW system, the design cost is greatly reduced. Experiments were carried out after theoretical explanation of focusing principle, micropillar design performance, and electrode design. Experiments show that our chip can achieve deflection separation of 20 μm particles at 24.95–41.58 MHz. At the frequency of 33.7 MHz, the transverse migration distance of 20 μm particles is the smallest, and the separation purity and efficiency are as high as 92% and 100%, respectively.

## 2. Materials and Methods

### 2.1. Design of the Microfluidic Separation Chip

**Figure 1:**
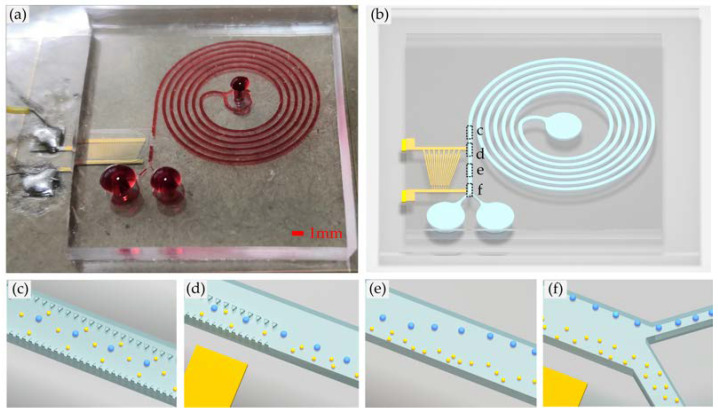
(**a**) Physical picture of the sorting chip; (**b**) Overall schematic diagram of the sorting chip; (**c**) The large particles are arranged in a line and the small particles are randomly scattered throughout the flow channel at the beginning of contact with the micropillar array; (**d**) All particles converge in the area 150 μm away from the outer wall of the flow channel when leaving the micropillar array region; (**e**) The large particles are deflected by acoustic control, while the small particles keep their original trajectory and continue to move forward; (**f**) Particles of different sizes are discharged from their respective outlets.

The proposed micro-device consists of the spiral channel and the TSAW separator. Polydimethylsiloxane (PDMS) was used to fabricate the microchannel. We plasma-bonded the PDMS to 128°Y-X piezoelectric lithium niobate (LiNbO3) crystal substrate patterned with a slanted gold IDT that generates the TSAW, as shown in Figure 1b. The physical picture of the sorting chip is shown in Figure 1a. When reaching the micropillar array at the exit, the large particles are arranged in a line, and the small particles are randomly scattered throughout the flow channel, as shown in Figure 1c. At the exit of the micropillar array, all particles converge in the area 150 μm away from the outer wall of the flow channel. The large particles are almost arranged in a line, and the small particles are randomly distributed, as shown in Figure 1d.

**Figure 2:**
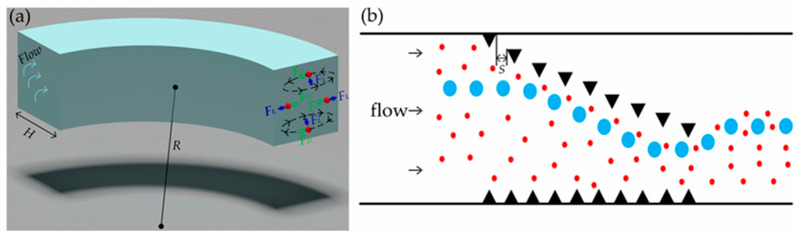
(**a**) The secondary flow in the curved channel forms two symmetrical vortices in which the particles are subjected to inertial lift *F_L_* and Dean drag *F_D_*; (**b**) The particles are deflected upward due to the sudden disappearance of the end pillar of the micropillar array.

In order to ensure the effect of acoustic waves on particles, a spiral flow channel with micropillars is introduced in front of the acoustic wave area. In the spiral channel, the radial pressure gradient caused by centrifugal force creates additional flows—secondary or Dean flows. The momentum of the fluid at the center line of the channel is higher than that of the wall, which drives the relatively stagnant elements near the channel wall to move inward along the circle, forming two converse vortex flows (Figure 2a). A particle in a curved channel with Reynolds number (*Re*) between 1 and 100 experiences both an inertial lift *F_L_* and a Dean drag *F_D_*. With the addition of secondary flow, the spiral flow channel can accelerate the particle focusing effect and help the particle to reach the equilibrium position faster. The final behavior of suspended particles is predicted by *R_f_*, which is denoted as Equation (1) [22]:

(1) $$R_{f}=\frac{a^{3}R}{H^{3}}$$

where *R* is the radius of curvature, *a* is the particle diameter and *H* is the flow channel width. The dependency of *R_f_* on the particle size implies that it is possible to separate the particles according to their size. In order to ensure that the spiral channel can achieve the focus of particles larger than 6 μm and ensure that the channel is not blocked, we set the channel width *w* and height *h* as 300 μm and 50 μm, respectively. The particle constraint inequality *λ* = *a*/*H* = a (*h* + *w*)/2*hw* > 0.07 is satisfied. We set the flow rate in the channel to 1.2 mL per hour; particle size-based separation failed to perform at this flow rate.

After passing through six spiral channels, the large particles are arranged in a straight line at the exit by inertial focusing. Due to the limitation of the average flow rate in the channel, the small particles cannot reach the focus and disperse evenly throughout the channel. Some randomly dispersed small particles will flow out of the target outlet and the purity of chip separation will decrease. To solve this problem, we designed the micropillar array in the spiral channel as shown in Figure 2b. The starting point of an offset micropillar array is set in the inner wall of the flow channel to ensure the convergence of dispersed particles in the flow channel. Considering that the sudden disappearance of the end pillar causes the particles to deflect upward, we set the end of an offset micropillar array in the area 100 μm away from the outer wall to achieve the particle aggregation within the area 150 μm away from the outer wall of the flow channel. The addition of an offset micropillar array results in the change of flow field, which may lead to the movement of some target particles close to the outer wall of flow channel. If target particles are close to the outer wall of the flow channel, they may enter the corner effect region. The target particles entering the corner effect region are not affected by the acoustic radiation force and thus flow out of the non-target outlet, which reduces the sorting efficiency of the chip. We designed a non-offset micropillar array on the outer wall of the flow channel to keep particles away from the corner effect region.

### 2.2. Separation Mechanism

In the TSAW separator, when the particle size is much smaller than the wavelength, the TSAW force *F*_TSAW_ controlling the particle movement can be calculated as Equation (2) [23]:

(2) $$F_{\mathrm{TSAW}}=-\pi a^{3}\left[\frac{2\kappa_{0}}{3}R_{e}\left(f_{1}^{*}P_{1}^{*}\nabla P_{1}\right)-\rho_{0}R_{e}\left(f_{2}^{*}v_{1}^{*}\nabla v_{1}\right)\right]$$

where *P*_1_ and *v*_1_ are the first-order acoustic pressure and velocity fields; *R_e_* denotes the real imaginary parts of fields; * stands for complex conjugation. Factors *f*_1_, *f*_2_, *γ* and ${\tilde{\delta }}_{v}$ are given by Equations (3)–(6), respectively [23].

(3) $$f_{1}=1-\frac{\kappa_{P}}{\kappa_{0}}$$

(4) $$f_{2}=\frac{2\left(1-\gamma \right)\left(\rho_{P}-\rho_{0}\right)}{2\rho_{P}+\rho_{0}\left(1-3\gamma \right)}$$

(5) $$\gamma =-\frac{3}{2}\left[1+i\left(1+{\tilde{\delta }}_{v}\right)\right]{\tilde{\delta }}_{v}$$

(6) $${\tilde{\delta }}_{v}=\frac{\sqrt{2\eta }}{\sqrt{\omega \rho_{0}}}$$

**Figure 3:**
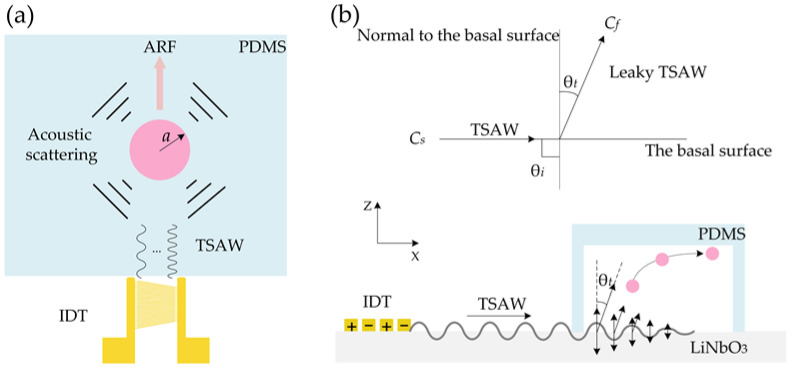
The interaction of TSAW propagating through a fluid with a suspended spherical particle. (**a**) Because of the scattering of TSAW waves on the surface of a spherical particle, ARF is generated and the direction is always the same as the propagation direction of the wave; (**b**) Complex fluid-structure coupling effect between TSAW wave and fluid results in TSAW wave leakage. The leakage TSAW wave propagates in the channel fluid at the Rayleigh angle (*θ_t_*), driving the particle to achieve the deflection function. The interaction of TSAW propagating through a fluid with a suspended spherical particle.

*κ*_0_ = 1/(*ρ*_0_c^2^_0_) and *κ_p_* denote the compressibility of the liquid and particle, respectively; *ρ*_0_ and *ρ_p_* are the density of liquid and particle, respectively; *w* is the angular frequency of excitation and *η* is the shear viscosity coefficient of the fluid. It can be obviously concluded that the *F*_TSAW_ is dependent on the radius, compressibility and density of particles, the compressibility and density of the liquid, and the angular frequency of excitation. Figure 3a depicts the interaction between sound waves and suspended particles in a fluid. When the dimensionless parameter *κ* = *k* × *a* = 2π/*λ_w_* × *a* (with the wavelength in water *λ_w_*, the particle radius *a*, and the wavenumber in water *k* ≥ 1), the sound wave exerts a net momentum transfer on the particles due to spherical anisotropic scattering, resulting in longitudinal migration of the particles (from bottom to top in Figure 3a). Particles undergo acoustic radiation force (ARF) during their longitudinal migration. ARF acting on suspended particles can be easily manipulated by adjusting TSAW frequency or changing particle diameter. The side view of the device in Figure 3b shows the complex fluid-solid coupling effects of TSAW entering the fluid. The coupling forms a leaking TSAW. The sound velocity on LiNbO3 substrate surface of 128°YX is *C_S_* = 3992 m/s, the incidence angle *θ_i_* is 90°. The angle of transmitted leaky TSAW (i.e., Rayleigh angle) may be found by Employing Snell’s law, *θ_t_* = sin^−1^(*C_f_/C_S_*) ≈ 21.8° [24]. Thus, particle deflection is divided into two processes. First, it is pushed along an inclined line with an angle of *θ_t_*; Second, the particles are near the top of the microchannel and move horizontally forward.

When particles enter the acoustic control region, the large particles can achieve deflection by adjusting the applied signal frequency, while the small particles keep the original motion track and continue to move forward, as shown in Figure 1e. At the outlet of the flow channel, the large particles flow out of the target outlet (upper outlet in Figure 1f) and the small particles flow out of the non-target outlet (lower outlet in Figure 1f).

A slanted IDT device which can excite multiple frequencies is used in this study. The transverse migration length required for 20 μm particle deflection will change with different frequencies. The use of slanted IDTs aims to obtain the optimal deflection frequency (minimum transverse migration distance required for deflection) for 20 μm particles. The electrode width is designed to be left (40 μm) and right (24 μm). Applying radio frequency (RF) signals of 24.95 to 41.58 MHz to IDTs produces TSAWs to deflect the large particles. It has been shown that polystyrene particles can achieve deflection when *κ* = 1.28 ± 0.20 [25]. At these frequencies, particles larger than 17 μm are deflected by acoustic radiation forces.

### 2.3. Fabrication of the Chip

**Figure 4:**
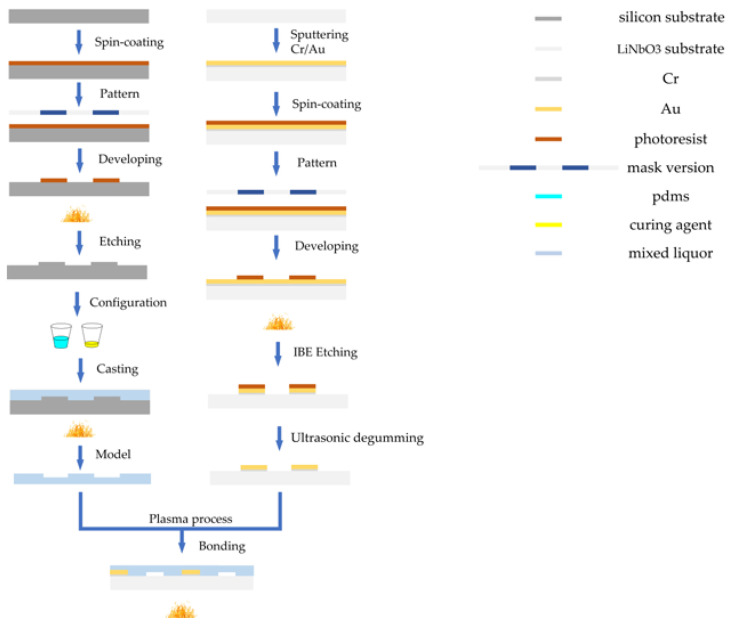
Flow chart of microfluidic chip manufacturing process.

The fabrication of a PDMS channel is mainly based on soft lithography technology, and the IDT electrode substrate adopts an ion beam etching (IBE) process. The production process is shown in Figure 4. First, a positive photoresist (AZ6130) was spun with a thickness of 2.5 μm onto a clean 4-inch silicon wafer. After baking for 1 min at 100 °C, the silicon mold was exposed to ultraviolet (UV) exposure. The exposed silicon mold was developed in ZX238 solution for 25 s and then baked (120°, 30 min). Subsequently, we placed the model in deep silicon etching equipment to etch at a depth of 50–52 µm. According to PDMS: curing agent = 10:1 ratio for configuration, mixed well and poured on the mold. The PDMS were peeled from the mold after hot drying at 75 °C for 40 min. The TSAW electrode substrate separator was fabricated by IBE etching process. After sputtering Cr/Au (20 + 100 nm) on lithium niobate substrate, photolithography was carried out. The lithography process was similar to the silicon process. After baking, the substrate was etching by IBE for 5 min to fabricate the IDT electrodes. Then, the etched model was put into acetone solution for ultrasonic degumming. After the surfaces of the PDMS channel and the LiNbO3 substrate were treated with a plasma process at 220 W for 15 s, they were aligned to achieve irreversible bonding. Finally, the bonded chips were baked at 85 °C for 30 min to enhance the bonding strength.

### 2.4. Sample Preparation and System Setup

Reagent 1 was configured with 20 µL 5 µm polystyrene fluorescent microsphere particle suspension (1 wt%) (5 μm particles show green under fluorescence excitation), 0.1 mL Tween and 4 mL deionized water. Reagent 2, containing 10 µm polystyrene fluorescent microsphere particle suspension (1 wt%) (10 µm particles show orange under fluorescence excitation), and Reagent 3, containing 20 μm polystyrene fluorescent microsphere particle suspension (1 wt%) (20 µm particles show blue under fluorescence excitation), were configured in the same way. The above three reagents were put into an ultrasonic oscillator for 5 min to make the particles evenly distributed in the reagents. The reagents were pumped into the microfluidics chip through a plastic hose (5 mL) with the help of a microinjection pump. The flow in the chip channel was achieved by adjusting the speed of the microinjection pump. A vector network analyzer was used to output a sine wave signal of a certain frequency. The particles were tracked by an optical microscope and a high-speed CCD camera to record the movements of the particles. A blood cell counter was used to measure the percentage of particles in solutions collected at different outlets. At least five sampling tests were required for each export.

## 3. Results

### 3.1. Simulation of Surface and Section Velocity Distribution in Microchannel

**Figure 5:**
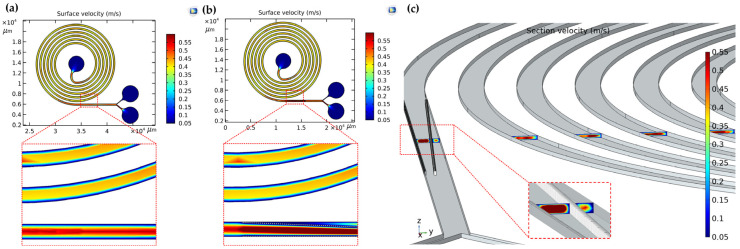
(**a**) The surface velocity distributions in the micropillar-free channel; (**b**) The surface velocity distributions in the micropillar channel; (**c**) The section velocity distributions of each return section in the micropillar channel.

When the sample flow is injected into the spiral channel at a certain speed, the particles will have different degrees of focusing due to the velocity limitation. Therefore, it is of great significance to study the flow velocity inside the flow channel. COMSOL software was used to simulate the surface and section velocity distribution of microfluidic chip to guide the experimental device. In order to verify the influence of micropillar arrays on secondary flow at constant flow rate, the velocity distributions in channels with and without micropillars are compared. In carrying out this work, we must take the influence of flow rate on acoustic control into account. The increase of velocity will shorten the time of particles movement in the acoustic interaction region and thus the effective driving of particles cannot be realized. Target particles may flow out of the non-target outlet and thus reduce the sorting efficiency of the chip. K. Mutafopulos et al. demonstrated effective acoustic manipulation at a flow rate of 25 μL/min [15]. The flow rate of the fluid was set at 20 μL/min in this research. The results show that the velocity in the center of the channel is higher than the other positions in the micropillar-free channel (Figure 5a). In the micropillar channel, the addition of micropillar arrays results in a change of flow field and an increase of flow field velocity along the micropillar offset direction (Figure 5b). This causes the particles in the fluid to move in the direction of the micropillar array.

Figure 5c shows the flow field distribution of each return section of the adopted channel with micropillars. It can be seen that there is a considerable velocity gradient in the center and wall of the parametric flow channel before the fluid reaches the micropillar array so as to ensure the formation of secondary flow. The formation of secondary flows will help particles reach their equilibrium position more quickly. When the fluid reaches the micropillar array, the fluid velocity is different on the left and right sides of the micropillar array. The fluid velocity on the left side of the micropillar is greater than that on the right side of the micropillar, as shown in the small illustration in Figure 5c. The micropillar structure concentrates particles in a specific region of the channel. Particle migration under offset micropillars has been tested and demonstrated several times below.

### 3.2. Related Performance Tests for Micropillar Arrays and Channel Outlets

The optimization of micropillar arrays is very necessary, because the parameters of micropillar arrays directly affect the aggregation performance of particles with various particle sizes. The test results show that particles will not pass through the micropillar gap when the micropillar gap is less than or equal to 4 times the particle radius. If the micropillar gap is larger than 4 times the particle radius, some of the small particles will pass through the micropillar gap and move against the inner wall of the flow channel, which will undoubtedly reduce the chip’s convergence performance and affect the subsequent sorting effect. The minimum particle diameter tested in this experiment is 5 μm, so the micropillar gap was set to 10 µm.

**Figure 6:**
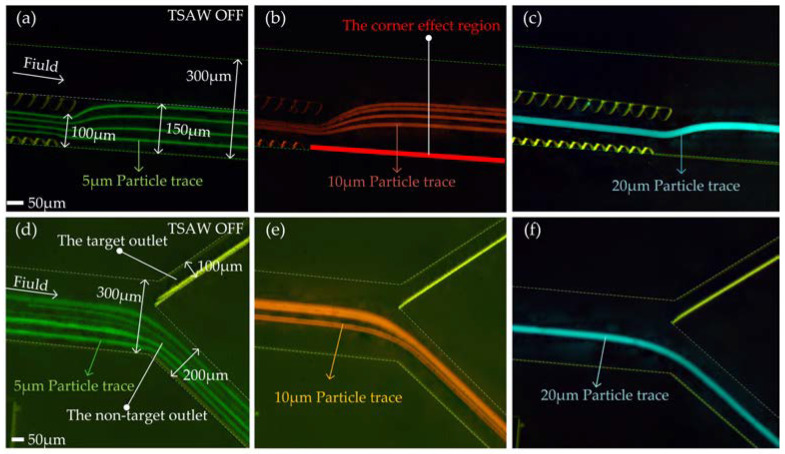
The test effect diagram after superimposing the particle tracks of each particle size: (**a**) 5 μm particle tracks test diagram at the end of micropillar; (**b**) 10 μm particle tracks test diagram at the end of micropillar; (**c**) 20 μm particle tracks test diagram at the end of micropillar; (**d**) 5 μm particle tracks test diagram at the outlet of the flow channel; (**e**) 10 μm particle tracks test diagram at the outlet of the flow channel; (**f**) 20 μm particle tracks test diagram at the outlet of the flow channel.

After the micropillar, all particles converge in the area 150 μm away from the outer wall of the flow channel. In order to realize that when TSAW is off, all particles flow out of the non-target outlet, and when TSAW is on, the target particles flow out of the target outlet and all other particles flow out of the non-target outlet, the proportion of the width of the outlet needs to be designed. We set the outlet width ratio as the target outlet (upper outlet in Figure 6d–f):non-target outlet (lower outlet in Figure 6d–f) = 1:2, that is, the target outlet and non-target outlet widths are 100 μm and 200 μm, respectively.

With TSAW off, we tested the distribution of Reagent 1 (containing 5 μm particles), Reagent 2 (containing 10 μm particles), and Reagent 3 (containing 20 μm particles) at the end of the micropillars and the outlets of the flow channel (Video S1). Figure 6a–f shows the test effect diagram after superimposing the particle tracks of each particle size. As can be seen from Figure 6a–c, at the end of the micropillar array, particle trajectories gradually change from multiple to one with the increase of the particle diameter. The 5 μm, 10 μm, and 20 μm particles are deflected to some extent at the end of the micropillar array, but they are all kept in the area 150 μm away from the outer wall of the flow channel. The 20 μm particles (target particles) were away from the corner effect region (highlighted in red in Figure 6b). In general, the addition of an offset micropillar array makes particles of all sizes converge within the area 150 μm away from the outer wall of the flow channel. The addition of a non-offset micropillar array keeps the target particles far away from the corner effect region. As can be seen from Figure 6d–f, 5 μm, 10 μm, and 20 μm particles all flowed out of the non-target outlet. This result indicates that the micropillar array structure designed by us can realize the convergence of particles with different particle sizes and is well matched with the spiral flow channel.

### 3.3. Acoustic Separation Performance Testing and Analysis

**Figure 7:**
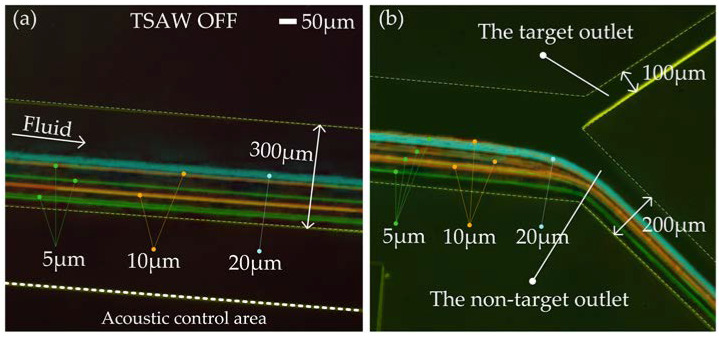
The test effect diagram after superimposing the particle tracks of each particle size: (**a**) Test diagram of the particle tracks in acoustic control area; (**b**) Test diagram of the particle tracks at the outlets of the flow channel.

We took 1.5 mL of Reagents 1, 2, and 3 to configure the sample solution. The distribution of particles in the acoustic separation region and at the outlet of the flow channel was measured under TSAW off and TSAW on modes, respectively. Figure 7a,b shows the test effect diagram after superimposing the particle tracks of each particle size. With TSAW off, 5 μm, 10 μm, and 20 μm particles did not deflect in the acoustic separation region, kept their original trajectory moving forward (Figure 7a, Video S1), and all flowed out of the non-target outlet (lower outlet in Figure 7b, Video S1).

**Figure 8:**
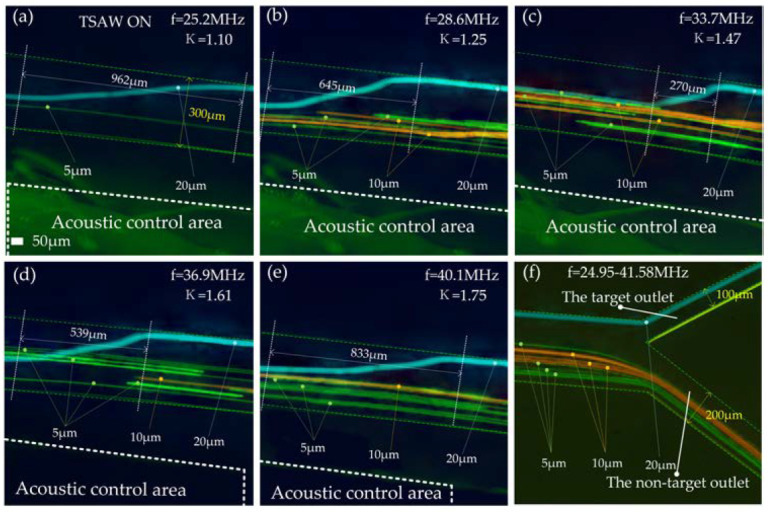
The test effect diagram after superimposing the particle tracks of each particle size: (**a**) Track test diagram of particles in the acoustic control region when *f* = 25.2 MHz; (**b**) Track test diagram of particles in the acoustic control region when *f* = 28.6 MHz; (**c**) Track test diagram of particles in the acoustic control region when *f* = 33.7 MHz; (**d**) Track test diagram of particles in the acoustic control region when *f* = 36.9 MHz; (**e**) Track test diagram of particles in the acoustic control region when *f* = 40.1 MHz; (**f**) Track test diagram of particles at the outlet of the flow channel when *f* = 24.92–41.58 MHz.

The electrode width range is 24–40 µm, and the corresponding excitation frequency range is 24.95–41.58 MHz. Within this excitation frequency range, the *κ* corresponding to 20 μm particles is 1.09–1.81. We tested the motion trajectory of 20 μm particles in the acoustic separation region at different frequencies (Figure 8a–e). In the figure, we give the transverse displacements required by particles during deflection at different frequencies. This shows that the 20 μm particles were deflected over the entire frequency range. When *f* = 33.7 MHz, *κ* = 1.47, the particle size of 20 μm has the smallest transverse displacement. After leaving the acoustic separation area, 20 μm particles move forward against the inner wall of the flow passage and flow out of the target outlet (upper outlet in Figure 8f). The 5 μm and 10 μm particles did not deflect when moving to the acoustic separation region, kept the original trajectory moving forward, and all flowed out of the non-target outlet (lower outlet in Figure 8f) in the whole frequency range (24.95–41.58 MHz). See Video S1 for the test video of the acoustic control area and channel outlet when *f* = 33.7 MHz.

### 3.4. Testing and Analysis of Separation Purity and Efficiency

**Figure 9:**
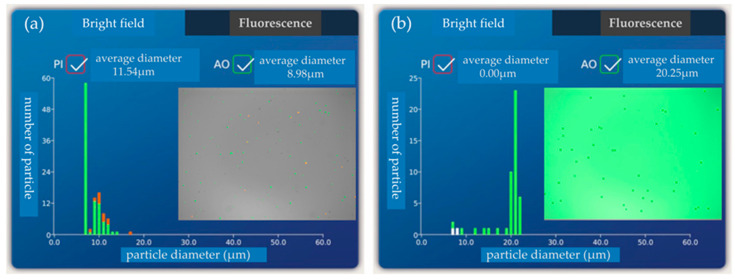
(**a**) 20 μm particle content test at the target outlet during the first sampling; (**b**) The 20 μm particle content test at the non-target outlet during the first sampling.

When *f* = 33.7 MHz, 20 μm particles flow out of the target outlet while 5 μm and 10 μm particles flow out of the non-target outlet. The percentage of 20 μm particles in the target outlet represents the purity of the separation chip. The separation efficiency of the chip is characterized by the percentage of 20 μm particles in the non-target outlet. Five samples were taken at the target outlet and non-target outlet, respectively, and then detected by a blood cell counter. Figure 9a,b for the 20 μm particle content test at the target outlet and non-target outlet during the first sampling are shown, respectively. There are no 20 μm particles in the non-target exit (Figure 9a), and only 20 μm particles in the target exit (Figure 9b).

We observed that a small amount of 5 μm or 10 μm particles were mixed in the target outlet, and the overall separation purity was more than 92%. There were no 20 μm particles in the non-target outlet, and the overall sorting efficiency of the chip is up to 100%, as shown in Table 1.

## 4. Discussion

We compare this chip with a spiral inertial separation chip [26] and the chip based on acoustic separation [15,16]. The comparison is shown in Table 2.

As can be seen from the data in Table 2, a spiral inertial separation chip [26] requires a higher flow rate. Under high flow-rate conditions, cell activity will be greatly reduced, even resulting in death. Because size-based particle separation is possible at high flow rates, additional sheath flows are not required. In the design of biological cell separation and detection, our chip is more able to meet the needs at hand. In addition, our chip is more than three times smaller than the inertial chip cascade deterministic lateral displacement (DLD) arrays. Compared with the other two chips based on acoustic separation [15,16], although the separation purity of our chip is not greatly improved, the separation efficiency reaches 100%. Most importantly, there are very few sorting chips in the current study that do not require an additional sheath flow. Our work improves this problem by achieving high separation purity and accuracy without sheaths, greatly reducing experimental complexity and the possibility of biological contamination.

## 5. Conclusions

This paper describes a microfluidic device based on TSAW which can collect 20 μm particles from the mixture of 5 μm, 10 μm, and 20 μm particles without a label or sheath. The device greatly improves the purity and efficiency of the separation chip by combining the spiral channel with micropillar arrays and a slanted gold IDT. The relationship between the maximum array clearance setting (Smax) and particle radius (*a*) is obtained through experimental tests: Smax = 4*a*. The array of micropillars arranged in this relation in the spiral channel allows the collection of particles to be independent of particle size. A micropillar array on the outer wall of the flow channel is proposed to keep the target particles away from the corner effect region. The separation chip designed by us achieves deflection separation of 20 μm particles at 24.95–41.58 MHz, and the optimal deflection of 20 μm particles is achieved at 33.7 MHz (*κ* = 1.47), with separation purity and efficiency as high as 92% and 100%, respectively. This work shows the improvement of particle convergence and particle separation. The design demonstrates the possibility of free regulation of particles with different particle sizes. In addition, the chip processing technology based on TSAW proposed in this work is expected to be used for clinical trial analysis.

## Figures and Tables

**Table 1:** Test statistics of particle content of each particle size in different outlets.

Outlet Type	Sample Number	20 μm Particle Concentration	5 μm/10 μm Particle Concentration	Total Particle Concentration	20 μm Particle Content Percentage
The target outlet	Sample 1	1.32 × 10^5^/mL	0.00 × 10^0^ mL	1.32 × 10^5^/mL	100%
	Sample 2	3.47 × 10^5^/mL	1.75 × 10^4^/mL	3.65 × 10^5^/mL	95%
	Sample 3	1.26 × 10^5^/mL	0.00 × 10^0^ mL	1.26 × 10^5^/mL	100%
	Sample 4	1.72 × 10^5^/mL	1.50 × 10^4^/mL	1.87 × 10^5^/mL	92%
	Sample 5	3.33 × 10^5^/mL	2.51 × 10^4^/mL	3.58 × 10^5^/mL	93%
The non-target outlet	Sample 1	0.00 × 10^0^ mL	3.07 × 10^5^/mL	3.07 × 10^5^/mL	0%
	Sample 2	0.00 × 10^0^ mL	2.48 × 10^5^/mL	2.48 × 10^5^/mL	0%
	Sample 3	0.00 × 10^0^ mL	3.42 × 10^5^/mL	3.42 × 10^5^/mL	0%
	Sample 4	0.00 × 10^0^ mL	1.78 × 10^5^/mL	1.78 × 10^5^/mL	0%
	Sample 5	0.00 × 10^0^ mL	2.55 × 10^5^/mL	2.55 × 10^5^/mL	0%

**Table 2:** Comparison list of different chips.

Chip Type	Flow Velocity	Separation Purity	Separation Efficiency	Chip Size	Require Sheath Flow
[26]	400 µL/min	93.59%	99.9%	6.5 cm × 2 cm	No
[15]	25 µL/min	90%	not mentioned	not mentioned	Yes
[16]	67.5 µL/min	92.7%	not mentioned	2 cm × 1 cm	Yes
Our work	20 µL/min	92%	100%	2 cm × 2 cm	No

## Data Availability

Not applicable.

## Supplementary Materials

The following supporting information can be downloaded at: https://www.mdpi.com/article/10.3390/bios12050325/s1, Video S1: Entire working video.
